# Attitude regarding electroconvulsive therapy among psychiatric patients

**DOI:** 10.1192/j.eurpsy.2023.576

**Published:** 2023-07-19

**Authors:** M. Ayub

**Affiliations:** Psychiatry, Pak International Hospital, Karachi, Pakistan

## Abstract

**Introduction:**

Electroconvulsive therapy (ECT) is one of the few non-pharmacological stimulation treatment which is cost effective, effecious and lifesaving in various psychiatric disorders. Although myths and misconceptions prevailed in a society undermine the usefulness of such treatment.

**Objectives:**

Attitude towards Electroconvulsive therapy (ECT) among psychiatric patients.

**Methods:**

It was a descriptive cross-sectional study conducted at the Department of Psychiatry and Behavioural sciences, Jinnah Postgraduate Medical Centre (JPMC), Karachi from 22-Oct-2019 to 21-April-2020 and a total of 250 psychiatric patients were enrolled.

Methode; Attitudes toward ECT were assessed using ECT attitude questionnaire6 (Annexure III). A 15 items questionnaire, each item has three alternatives based on which responses were categorized into positive, negative, or ambivalent attitudes. Mean score was calculated for each.

Patients who were given 8 positive answers out of 15 were labeled as having a positive attitude. Patients who were given 8 negative answers out of 15 were labeled as having a negative attitude. Patients who were given 8 I don’t know answers out of 15 were labeled as having ambivalent attitude.

**Inclusion Criteria:**

Age 18-65 years

Either gender

Psychiatric patients, having awareness regarding their nature of illness and could give consent for study.

Patients with duration of illness >3 months.

**Exclusion Criteria:**

Psychiatry patients who have no awareness regarding their illness.

Patients with impaired cognitive

**Results:**

Forty-four (45.83%) patients had positive attitude, 36 (37.50%) had negative attitude and 16 (16.67%) had ambient attitude.

Further stratification was also performed on the basis of educational status, occupational status, duration of illness, psychiatric diagnosis, and previous experience of ECT. There was no significant association was found of these variables with attitude regarding ECT.

Mean age was 39.58±12.48 years included in this study. There were 55 (57.29%) female and 41 (41.71%) male patients. There were 72 (75.00%) patients were household workers, 04 (4.17%) students, 06 (5.25%) unskilled labour, 3 (3.13%) skilled labour, 10 (10.42%) professionals and just 01 (1.04%) were law enforcement worker. 19 (19.79%) patients were diagnosed with schizophrenia, 62 (64.58%) were diagnosed with unipolar depression and 15 (15.63%) were diagnosed with bipolar disorder. Source of ECT information was 11 (11.46%) electronic media, 09 (9.38%) print media, 19 (19.79%) social media and 57 (59.38%) was from health professionals. Forty-four (45.83%) patients had positive attitude, 36 (37.50%) had negative attitude and 16 (16.67%) had ambient attitude.

**Conclusions:**

Knowledge regarding electroconvulsive therapy (ECT) was low in psychiatric patients in Pakistan. Only 45.83% patients showed positive attitude towards ECT.

**Reference(s):**

Carney S, Geddes J. Electroconvulsive therapy. Br Med J. 2003;326:1343-4

Gangadhar BN., Kapur RL., Kalyanasundaram S. Comparison of electroconvulsive therapy with imipramine in endogenous depression: a double blind study. Br J Psychiatry.1982;141:367–71.

Kellner CH., Fink M., Knapp R., Petrides G., Husain M., Rummans T., et al. Relief of expressed suicidal intent by ECT: a consortium for research in ECT study. Am J Psychiatry. 2005;162:977–82.

Baghai T C, Moller HJ. Electroconvulsive therapy and its different indications. Dialogues Clin Neurosci. 2008 Mar; 10(1): 105–17.

Weiner RD., Coffey CE., Folk J., Fochtmann LJ., Greenberg RM., et al. American psychiatric association committee on electroconvulsive therapy, The practice of electroconvulsive therapy. 2nd ed. Washington, DC: American Psychiatric Association; 2001

**Disclosure of Interest:**

None Declared

